# Lexical Features of Economic Legal Policy and News in China Since the COVID-19 Outbreak

**DOI:** 10.3389/fpubh.2022.928965

**Published:** 2022-07-01

**Authors:** Jiaqi Liu

**Affiliations:** School of International Studies, Zhejiang University, Hangzhou, China

**Keywords:** lexical feature, corpus studies, news text, economic legal policy, COVID-19

## Abstract

Lexical features are influenced by different languages and genres. The study of lexical features in different genres of texts on the same topic is helpful to understand the universalities and peculiarities of languages. This study constructs a research on the lexical feature and word collocations of two self-build corpora (China's economic Legal Policy Corpus and English News Corpus during the COVID-19 pandemic), the methods of Quantitative Linguistics and context interpretation are adopted. It was found that: (1) the word length, word frequency, word cluster and high frequency word distribution in English economic news and Chinese economic legal policies are influenced by language and genre to some extent, and they conform to different functional image distribution; (2) during the COVID-19 pandemic, “development” has been the focus of China's economic legal policies and English news, the two have attached importance to economic recovery and taken a positive attitude toward it in different ways. These findings suggest that: (1) There are some universalities and peculiarities between English economic news and Chinese economic legal policies in the distribution of lexical feature; (2) there is a certain synchronization between laws and news, and both of them maintain a positive and objective attitude toward the economic development during the pandemic. This study carries out a macroscopic investigation on internal structure and external interpretation, which enriches the study on lexical features and cultural features of language and provides some references for relevant studies.

## Introduction

The study of language can be divided into two fields: the study of language structure and the study of language application [(1), p. 1], corpus plays an important role in language research in both fields. “As one of the branches of Computational Linguistics, Corpus Linguistics deals with the principles and practice of using corpora in language study. A corpus is a collection of linguistic data, either compiled as written texts or as a transcription of recorded speech” [(2), p. 413]. Corpus Linguistics takes corpus as the starting point of language description or uses corpus as the method to verify linguistic hypotheses (3). Halliday (4) believes that Corpus Linguistics links data collection with theoretical construction and promotes people's understanding of language.

Corpus Linguistics is widely used in the field of language teaching (5–7), translation studies (8–12), second language acquisition (13–15), discourse analysis (16–18). Through corpus analysis, effective comparison and data statistics can be made between the two or more corpora to better explain language phenomena.

From the perspective of Quantitative Linguistics, “Quantitative Linguistics concerns itself with the various language phenomena, language structures, structural properties, and their interrelations in real-life communicative activities,” through mathematical quantitative methods, “it conducts accurate measurement, observation simulation, modeling and explanation of these phenomena in order to discover the mathematica laws underlying the language phenomena” (19). To investigate the progress of discourse research at home and abroad on the basis of Quantitative Linguistics is an important reference to clarify the research context at home and abroad (20). Through the research method of Quantitative Linguistics, we can better describe the laws of language phenomenon and explain the framework of its internal mechanism.

Sinclair [(21), pp. 131–142] proposed lexical grammar under the influence of different languages and themes, held that word was the starting point of building lexical models, and proposed the framework of lexical semantic meaning relationships, the first of which was lexical collocation relations. Lexical features include word length, word frequency, high frequency word and others. Genre, language style, language and other factors that may affect lexical features and lexical collocation. Word is one of the important elements of text, the analysis of lexical features can better understand the differences between different genres of text. In the field of second language acquisition, there are many research achievements in lexical features (22–24). Lexical features are also the focus in the field of translation (25, 26).

In addition, context interpretation can also be used to interpret lexical features. Malinowski put forward “situational context” and “cultural context” respectively in 1923 and 1935. Up till now, after continuous development, the context theory has been applied in linguistics, the interpretation and restriction effects of context on the meaning of language have been recognized. Context can help us to understand the meaning of language in communication, stimulate the internalized language form, and promote effective and correct expression.

There is also much discussion about the universalities and peculiarities of languages. “What is the relationship between languages, are they fundamentally different from each other or do they have a common law? If they have anything in common, how much? These questions have always been the subject of concern and the focus of linguists” (27). At present, there are few comparative studies of different genres based on Quantitative Linguistics and corpus methods. During the pandemic, the country's legal policies can better reflect the country's response, and the news can also reflect a certain response attitude, so in order to better explore the differences between different genre text and understand the universalities and peculiarities of languages, this paper constructs a research on the lexical feature and word collocations of two self-build corpora (China's economic Legal Policy Corpus and English News Corpus during the COVID-19 pandemic), the methods of Quantitative Linguistics and context interpretation are adopted. It is hoped that this study may provide some tentative answers to the issue above by focusing on the following questions:

(1) What is the distribution characteristics of lexical features of legal language and news language? Do they follow certain laws or models?
(2) Whether the focus of China's economic laws and news during the COVID-19 pandemic are synchronized? What values do they reflect?

Question (1) aims to discuss the different distribution characteristics of the two texts at lexical level, and find out the commonness of the lexical features of the two texts. Question (2) is intended to explore whether there is a social and semantic connection between the two, and find out the values behind it. It is hoped that these questions can give some enlightenment to the comparison of words of texts in different genres with the same theme.

## Materials and Methods

Detailed information about the study's methods and materials, including data sources, procedures and data analysis are presented in this part.

### Data Sources

The construction of Legal Policy Corpus (LPC) and News Corpus (NC) is the foundation of this research, which needs to determine representativeness of corpora and collect data.

Legal policies include not only laws, but also administrative regulations, judicial interpretation, departmental rules, military regulations, party regulations, group provisions, industry regulations and other different types of legal provisions. There are also differences in the writing criteria and style among different types of legal provisions. Laws, administrative regulations, judicial interpretation and departmental rules are important parts of legal policies, which have distinct linguistic features and good representativeness. The outbreak of COVID-19 pandemic is sudden and uncertain, and the legal policies issued by the government during this period are of good reference significance for the study of legal language. The COVID-19 pandemic is having a serious impact on various aspects of the country, especially the economy, which is an area of national priority and urgent need to restore order. Beida Fabao-Laws & Regulations Database developed by Peking University on the basis of its Legal Information Center in 1985, after more than 30 years to improve and perfect, it is one of the domestic advanced mature professional database of laws and regulations. Therefore, in order to objectively reflect the facts of the legal corpus and investigate the linguistic characteristics of the legal text, this study selects the Beida Fabao-Laws & Regulations Database as the data source of the Legal Policy Corpus (LPC).

China Daily is one of the six national key media websites and a comprehensive news media website integrating news information and entertainment services. It serves the domestic and international mainstream medium and high end readers, and is the online bridge between China and the world. As the largest English news portal in China, China Daily is one of the national key news websites. It presents every process of China's development to the world with objective and accurate reporting, unique news perspective and humanized expression. China Daily has carried a lot of reports on the COVID-19 pandemic, including detailed reports on the economy, politics and culture of countries around the world during the pandemic. It has also tracked China's response policies and their effects in detail. Therefore, in order to objectively reflect the facts of news corpus and investigate the linguistic features of news texts during the COVID-19 pandemic, China Daily was selected as the data source of News Corpus (NC) in this study.

### Produce

In this study, economic legal policies issued in 2020 were retrieved through Beida Fabao-Laws & Regulations Database (interview date: November 4, 2020). Laws and regulations that have expired or have no relation to the COVID-19 pandemic and economy are removed. Finally, the current economic legal policies in effect during the pandemic from January 2020 to November 2020 will be retained. A total of 39 pieces were collected, including four laws, three administrative regulations, three judicial interpretations, and 29 departmental rules, with a total word count of 174,904 in Chinese (see Table 1).

**Table 1:** Basic information of the two corpora.

**Corpus**	**Type**	**Language**	**Words**	**Timespan**	**Validity**	**Source**
Legal Policy Corpus (LPC)	Official	Chinese	174,904	Jan.2020—Nov.2020	Currently effective	https://www.pkulaw.com/
News Corpus (NC)	Official	English	123,333	Apr.2020—Nov.2020	International section	http://www.chinadaily.com.cn/

At the same time, this study searched the economic news after 2020 with “COVID-19” as the keyword on China Daily website, and manually excluded the news irrelevant to domestic economic policies. Finally, 202 English news with a total of 123,333 English words have been published on the international section from April 2020 till now (November 2020) were collected (see Table 1).

After collected the data, all of them were faithfully kept in a TXT format. Before data analysis of Chinese corpus, word segmentation is needed, and different word segmentation standards will lead to slight differences in statistical results. This study takes the above two corpora as the main materials, and uses ROST Content Mining System 6.0 word segmentation tool to do the word segmentation to the Chinese corpus and convert it into Unicode encoding.

After automatic processing, this paper manually proofreads and corrects the text to eliminate unnecessary punctuation marks or links, so as to increase the analyzability of the corpus.

AntConc 3.4.4w and Wordsmith Tool 6.0 were used for data analysis when analyzing Chinese and English corpus in this study. At present, there are many retrieval tools of corpus. AntConc 3.4.4W is adopted because it can provide retrieval function for Chinese corpus and can retrieve specific information of corpus to provide detailed data, and Wordsmith Tool 6.0 can supplement with it. AntConc 3.4.4w has functions such as Concordance Tool, Concordance Plot Tool, Clusters/N-Grams, Word List, etc. It can be used together with Wordsmith Tool 6.0 and they can be complementary to each other, enabling more accurate retrieval of Chinese and English corpora and statistics of word frequency and collocation. At the same time, adopt the method of Quantitative Linguistics, correlation analysis of word length and word frequency was carried out by IBM SPSS Statistics 23, and matching analysis of word length and word frequency dispersion point graph and function model was carried out by RStudio V0.99.902.

### Data Analysis

Vocabulary is the basic unit of language structure and plays a fundamental role in language performance. In traditional linguistic studies, vocabulary is considered to be lawless [(28), p. 3]. In recent years, vocabulary has gradually become the main object of linguistic research. Sinclair (29) believed that the rise of vocabulary research was mainly due to Halliday's research methods and the development of computers. Halliday (30) proposed the importance of vocabulary in the study of grammar and highlighted the position of corpus. The rise of computers makes it possible to further analyze the vocabulary and sentences of large-scale corpora. As one of the branches of computational linguistics, “corpus linguistics deals with the principles and practice of using corpora in language study,” “a corpus is a collection of linguistic data, either compiled as written texts or as a transcription of recorded speech” [(2), p. 413]. With the appearance of corpus linguistics, grammatical relations at the lexical level based on a large number of natural texts can be explored.

This paper compares China's economic legal policies and economic news during the COVID-19 pandemic, and discusses the differences between legal policies and news in terms of word length, high frequency words and their collocations. The basic information of the two corpora in this study is as follows:

As shown in Table 2, the Type-Token Ratio (TTR) and the Standard Type-Token Ratio (STTR) of News Corpus were lower than those of the Legal Policy Corpus, while the mean word length and the mean sentence length were higher than those of the Legal Policy Corpus. TTR and STTR are commonly used in corpus linguistics to compare changes in lexical density. The lexical density of News Corpus is lower than that of the Legal Policy Corpus, but the mean sentence length and mean word length are higher than that of the LPC. It can be preliminarily speculated that compared with the legal policy, news tend to use more language units to express meaning with simplified vocabulary and low sentence complexity, which is also influenced by the differences in the types of Chinese and English. For example, in Chinese, verbs can be used together, while in English, there is always only one predicate verb in a sentence. For another example, Chinese predicate verbs have no tenses, person and number changes, but English predicate verbs have abundant changes, etc., which will more or less affect the comparative study of words in this paper. However, this paper mainly compares word length, word frequency, word clusters, high-frequency words and their collocations, rather than grammar and other language areas with great differences. In order to explore the lexical features of legal texts and news texts, the next section will analyze the word length and frequency, word cluster, high frequency words and their collocations in the two corpora.

**Table 2:** Basic features of the two corpora.

	**Legal Policy**	**News**
	**Corpus (LPC)**	**Corpus (NC)**
Tokens	90,648	122,271
Types	7,116	8,769
Type-token ratio (TTR)	7.85%	7.17%
Standard type-token ratio (STTR)	44.83%	44.06%
STTR standard deviation	52.29	54.83
STTR basis	1,000	1,000
Mean word length	1.87	5.20
Mean word length standard deviation	0.61	2.78
Mean sentence length	22.26	25.70

## Results

### Word Length

Zipf discovered the relationship between the occurrence frequency of words in the text and their frequency rank (serial number), and proposed a mathematical formula to describe this functional relationship, namely Zipf's law (31). Zipf [(32), p. 38] pointed out that the word length is usually inversely proportional to its frequency, and the relationship between the two is not completely proportional, but may conform to the non-linear mathematical function. The word length can reflect the degree of difficulty of the text, so as to reflect the complexity of language units. Human cognitive system and brain information processing mechanism makes speakers tend to choose short and simple words to express specific meaning in order to save energy consumption, which leads to the increase in the frequency of using shorter words in discourse (33). However, there are few studies on the distribution characteristics of word length in legal texts and news texts. Therefore, this section will compare the characteristics of word length in Chinese legal texts and English news texts to get a better idea of their textual complexity.

As shown in Table 2, the mean word length of English news corpus is higher than that of Chinese law corpus. Table 3 shows the basic information of the word length distribution of the two corpora. Chinese corpus calculates the word length with Chinese characters, while English corpus calculates the word length with letters.

**Table 3:** Word length distribution of legal policy corpus (LPC) and news corpus (NC).

	**LPC**	**NC**
1-letter words	21,859	2,484
2-letter words	64,514	18,905
3-letter words	5,533	21,371
4-letter words	1,601	18,023
5-letter words	130	12,607
6-letter words	48	11,505
7-letter words	1	12,184
8-letter words	3	9,604
9-letter words	0	6,395
10-letter words	0	4,532
11-letter words	3	2,831
12-letter words	1	1,257
13-letter words	0	1,195
14-letter words	0	291
15-letter words	0	104
16-letter words	0	28
17-letter words	0	9
18-letter words	0	3
19-letter words	0	0
20-letter words	0	5

As shown in Table 3, the relationship between word length and word frequency in the two corpora is in line with the economic principles of the language as a whole. In the Chinese Legal policy corpus (LPC), the proportion of 2-letter words is the highest, followed by the proportion of 1-letter words, which conforms to the main characteristics of the distribution of disyllable word length in Chinese and English (34). Apart from stylistic differences, Chinese and English also have great differences in types, so word length data of English News Corpus (NC) cannot be comprehensively compared with Chinese LPC data in all aspects. In order to understand whether there are similarities between the two corpora in terms of word length and word frequency, this section focuses on the analysis of their relevance. The Kendall's tau-b coefficient of rank correlation test by IBM SPSS Statistics 23 software showed that the word length distribution of LPC and NC was significantly correlated (*p* < 0.001), as Table 4.

**Table 4:** Correlation analysis of word length and frequency distribution between LPC and NC.

			**LPC**	**NC**
**Kendall's tau-b**	**LPC**	Correlation Coefficient	1.000	0.661**
		Sig. (2-ailed)	.	0.000
		N	20	20
	**NC**	Correlation Coefficient	0.661**	1.000
		Sig. (2-ailed)	0.000	.
		N	20	20

**. Correlation is significant at the 0.01 level (2-tailed).

**Figure 1:**
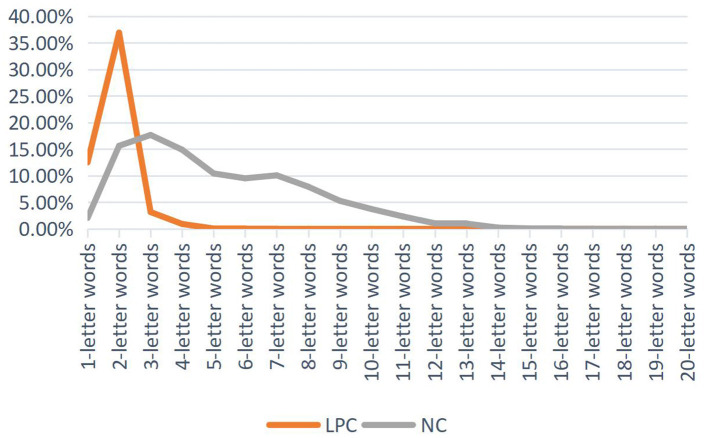
Word length percentage distribution of LPC and NC.

As shown in Table 4, on the whole, the distribution of word length and word frequency of Chinese LPC and English NC is significantly correlated, but there are still differences in the distribution of word length and frequency. LPC has more words with three letters and below, while NC has more words with three letters to ten letters than LPC, as shown in Figure 1.

Figure 1 is the word length percentage distribution of LPC and NC, which reflects the Principle of Least Effort. However, there are some differences between them. The percentage of 1-letter words and 2-letter words in LPC is higher than that in NC, and the percentage of 2-letter words occupies the highest proportion in LPC. Starting from 2-letter words, the distribution ratio of word length in LPC showed a downward trend, with no fluctuation, and the percentage of the 3-letter and above words tended to 0. In NC corpus, the proportion of 3-letter words is the highest, which shows an increasing trend before the 3-letter words. Starting from the 3-letter words, the distribution of the word length shows a declining trend with no obvious fluctuation amplitude, and shows a steady downward trend since the 7-letter words.

**Figure 2:**
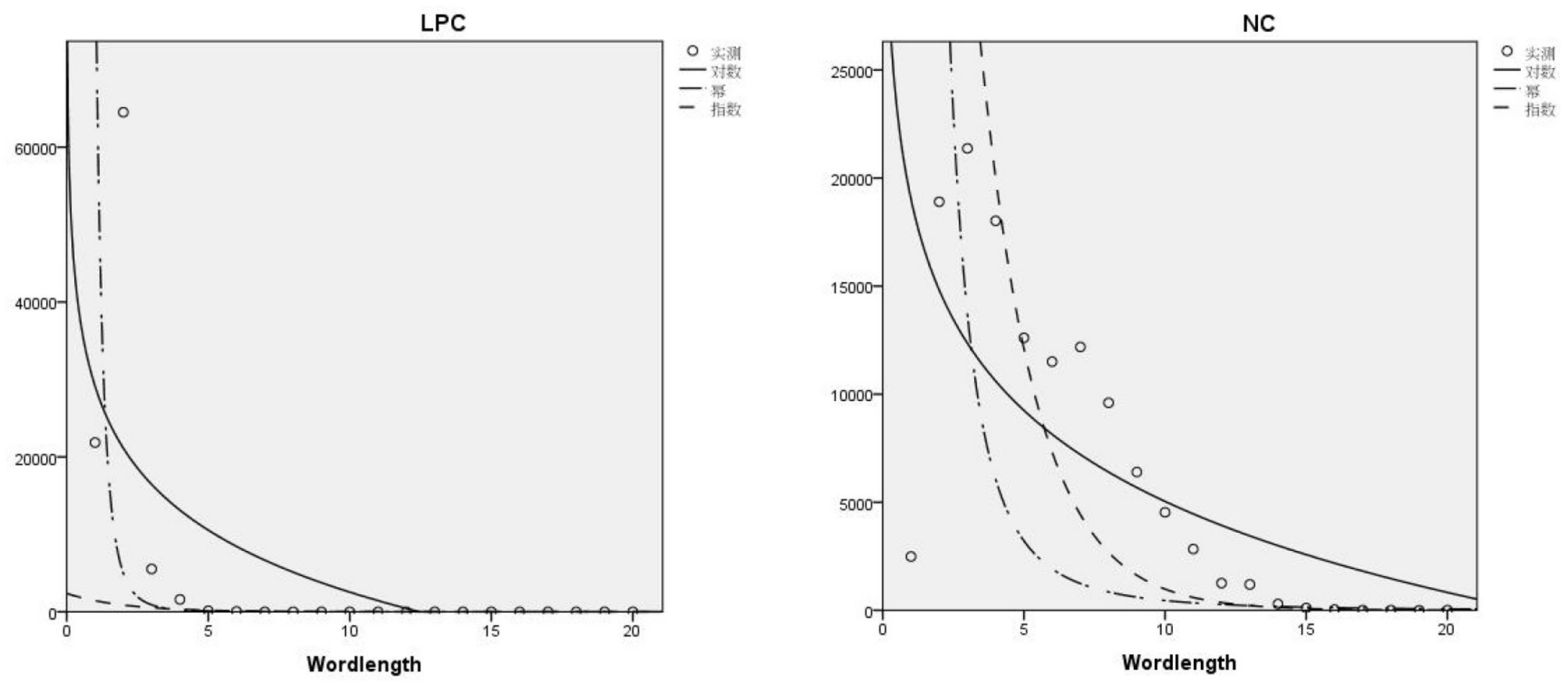
Dispersion diagram of word length and frequency of LPC and NC.

There have been many advances in the research on the relationship between word length and word frequency. For example, Zipf (32) proposed that the relationship between word length and word frequency is inversely proportional; (35) discussed the word length of Slovak poetry; (36) conducted a diachronic study on the distribution of Chinese word length; (37) discussed the influence of sentence length on the dependence distance, etc. As for the functional relation between word length and word frequency, (33) pointed out that the power function model could describe the regularity of Chinese words well. This section will discuss the functional relation between word length and word frequency in Chinese LPC and English NC, as shown in Figure 2.

Curve fitting was performed by SPSS, and the results were shown in Figure 2, the word length distribution of the two corpora can be seen intuitively. Among them, the word length distribution of Chinese LPC is closer to the power function distribution, and that of English NC is closer to the logarithmic function distribution. Compared with English NC, the distribution of word length and frequency in Chinese LPC is relatively discrete.

Tables 5, 6 are the basic information of model summary information and parameter estimation values of the logarithmic function, power function and exponential function of LPC and NC, respectively. Goodness of fit is an important indicator to judge whether a group of data conforms to a model. Through the curve fitting tool of SPSS, *R*^2^ can be used to judge the fitting effect of two groups of data. The value of *R*^2^ is between 0 and 1, when *R*^2^ > 0.75, it means that the data is applicable to this model, and the closer the value is to 1, the higher the fitting degree will be. For LPC, *R*^2^ in the power function model is 0.831 > 0.75, which conforms to the power function distribution. In the logarithmic function model and exponential function model, *R*^2^ <0.75, so it does not conform to the logarithmic and exponential function distribution; For NC, *R*^2^ > 0.75 in the exponential function model, but in the logarithmic and power function model, *R*^2^ <0.75, so it conforms to the exponential function distribution, but not to the logarithmic and power function distribution.

**Table 5:** Model summaries and parameter estimates of LPC.

**Dependent variable: LPC**							
**Equation**	**Model summaries**					**Parameter estimates**	
	***R*** ^2^	**F**	**df1**	**df2**	**Sig**.	**Constant**	**b1**
Logarithmic	0.396	11.783	1	18	0.003	29141.417	−11553.425
Power	0.831	88.799	1	18	0.000	91851.245	−4.206
Exponent	0.621	29.542	1	18	0.000	2366.724	−0.500

*Independent variable: Wordlength*.

**Table 6:** Model summaries and parameter estimates of NC.

**Dependent variable NC**							
**Equation**	**Model summaries**					**Parameter estimates**	
	***R*** ^2^	**F**	**df1**	**df2**	**Sig**.	**Constant**	**b1**
logarithmic	0.469	15.880	1	18	0.001	19032.511	−6078.008
power	0.487	17.092	1	18	0.001	309632.055	−2.838
exponent	0.810	76.784	1	18	0.000	149502.906	−0.503

*Independent variable: Wordlength*.

In general, the power function model can better explain the distribution characteristics of word length and frequency in Chinese LPC, while the exponential function model can better explain the distribution characteristics of word length and frequency in English NC. This can also be explained that the distribution characteristics of legal language and news do follow certain laws or models.

### Word Cluster

Lewis (38) believes that traditional grammar and vocabulary are not the basic structure of a language, but word cluster, which is “a kind of language structure with both lexical and grammatical features” (39). Word clusters can be composed of multiple words, which are not necessarily complete in structure and meaning. However, they have specific discourse functions and play an important role in language output, which can reflect the characteristics of language repetition. Both the language of law and the language of news are normalized and stylized to some extent. In this section, the word clusters of 2–7 words in the two corpora are taken as the research objects to observe their word cluster characteristics.

**Figure 3:**
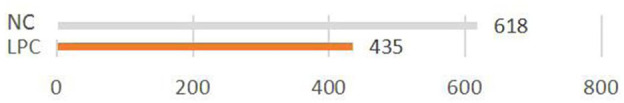
Word clusters of 2 to 7 words with frequencies over 20 in two corpora.

As shown in Figure 3, there is a certain difference in word clusters of 2 to 7 words which frequency exceeds 20 in the two corpora. Although LPC corpus has more words than NC, the number of word clusters with frequency over 20 in LPC is lower than NC. Next, this paper makes further statistics on the detailed data of word clusters of 2 to 7 words which frequency exceeds 10, as shown in Table 7.

**Table 7:** Word clusters information of 2 to 7 words with frequencies over 10 in two corpora.

	**LPC**	**NC**
word clusters of two words	854	1,298
word clusters of three words	299	388
word clusters of four words	175	103
word clusters of five words	117	34
word clusters of six words	92	10
word clusters of seven words	72	2
Total	1,609	1,835

**Figure 4:**
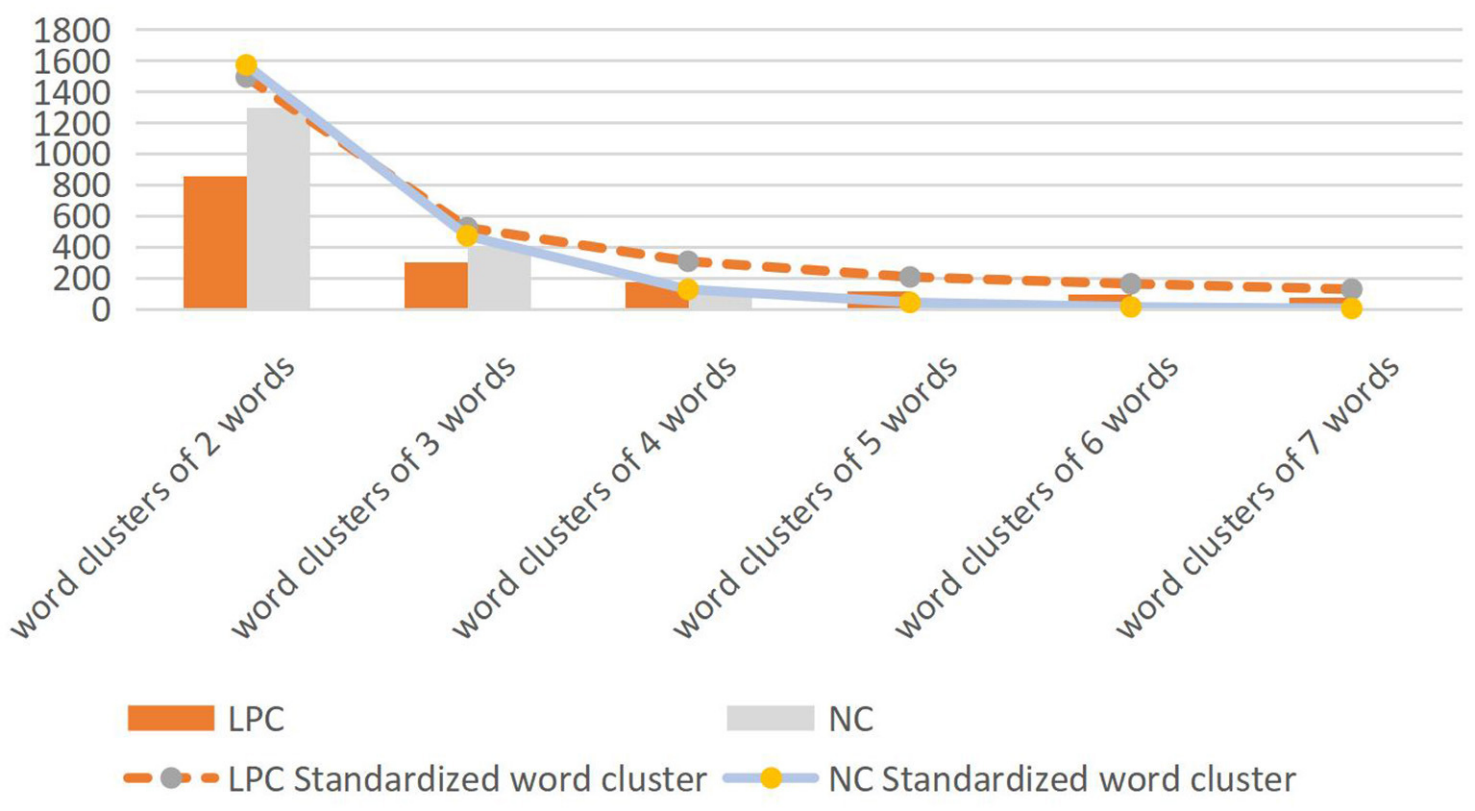
The number of word clusters and the distribution of standardized word clusters.

As shown in Table 7, the number of word clusters of 2–7 words and the total word clusters in Chinese LPC is lower than that in English NC, and the number of word clusters in both of them is concentrated in 2–4 words. The more words in the word cluster, the less of the word cluster. According to Xiao [(40), p. 74], standardized word clusters (word clusters/million word times) can be used to measure and compare the distribution of word clusters. Since the size of the two corpora in this study is about 100,000 words, standardized word clusters (word clusters/per 100,000 words) are adopted to measure in this section, as shown in Figure 4.

Word cluster is one of the effective means to explore textual features and judge text changes. As shown in Figure 4, the overall distribution of the two corpora is consistent, but there are also some changes and overlaps. The distribution proportion of NC word cluster in two words is higher than that in LPC, but the distribution proportion of other words is lower than that in LPC. In LPC, the number of word clusters of 2–3 words was lower than that of NC, but the number of word clusters of 4–7 words was higher than that of NC, and the gap is increasing. It can be seen that English news texts tend to use shorter word clusters compared with legal policy texts. Compared with news texts, legal policy texts are more likely to have repetitive language fragments or policy names, and Figure 4 also reflects the high repetition rate of long language fragments containing content words in Legal Policy Corpus. Next, this paper will discuss the distribution information of high frequency words of the two corpora in order to further compare their lexical features.

### Distribution Information of High Frequency Words

High frequency word is an important factor to measure language features. It not only reflect text features at the lexical level, but also reflect the areas that a text emphasizes to a certain extent, so as to reflect the focus at the semantic level. Wu (41) conducted a quantitative analysis of high frequency words from the perspective of literature year distribution and high frequency word distribution, and believed that the study of high frequency words was conducive to the integrated analysis of information resources; (42) studied the relationship between recognition of high frequency words from speech and second language (L2) listening comprehension, and believed that high frequency words have important research significance in the field of language acquisition. This section firstly takes Laciosa's definition of high frequency words (the lexical items appear at least 0.10%) as the standard [(43), p. 12], and carries out statistical analysis on the overall characteristics of high frequency words in the two corpora, as shown in Table 8.

**Table 8:** High frequency word distribution basic information of LPC and NC.

	**LPC**	**NC**
Number of high frequency words	61	113
Token Ratio of high frequency words	24.72%	50.38%
Type Ratio of high frequency words	0.86%	1.29%

As shown in Table 8, The number of high frequency words in English NC is more than that in Chinese LPC, and in English NC, the proportion of the token of high frequency word in the total token of the corpus is significantly higher than that in Chinese LPC. High frequency words show a high frequency in English corpus. From the perspective of practical application, the language of news is more active and covers a wide range than legal provisions; in terms of language categories, English has more morphological and lexical changes than Chinese. In order to further investigate whether the number of high frequency words affected by genre, on the basis of [(40), p. 67] statistical method to analyze high frequency words, ultra high frequency word (≥0.5% of total corpus), high frequency word (≥0.07% of total corpus), high frequency word (≥0.05% of total corpus), secondary high frequency word (≥0.03% of total corpus) and secondary high frequency word (≥0.02% of total corpus) of the two corpora will be further investigated, in order to increase the accuracy of the high frequency word distribution information.

**Figure 5:**
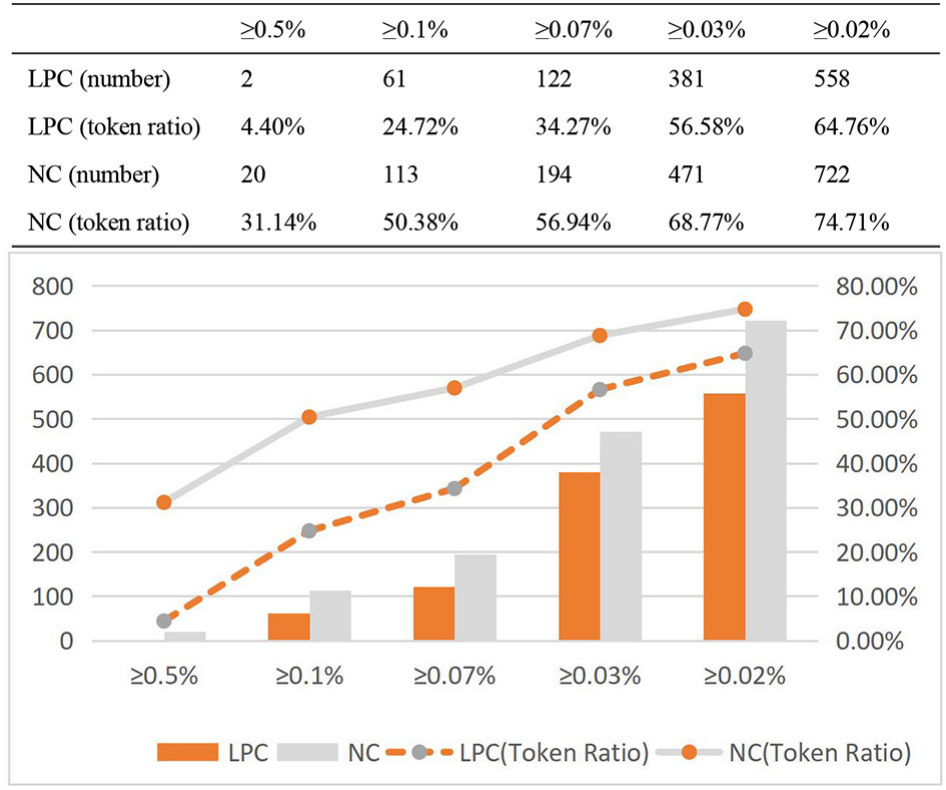
Token ratio distribution of high frequency words of LPC and NC.

As shown in Figure 5, the number of high frequency words in Chinese LPC is lower than that in English NC in all categories, and the token ratio of high frequency words in English NC is higher than that in Chinese LPC, and the token ratio of high frequency words of both LPC and NC is on the rise.

**Figure 6:**
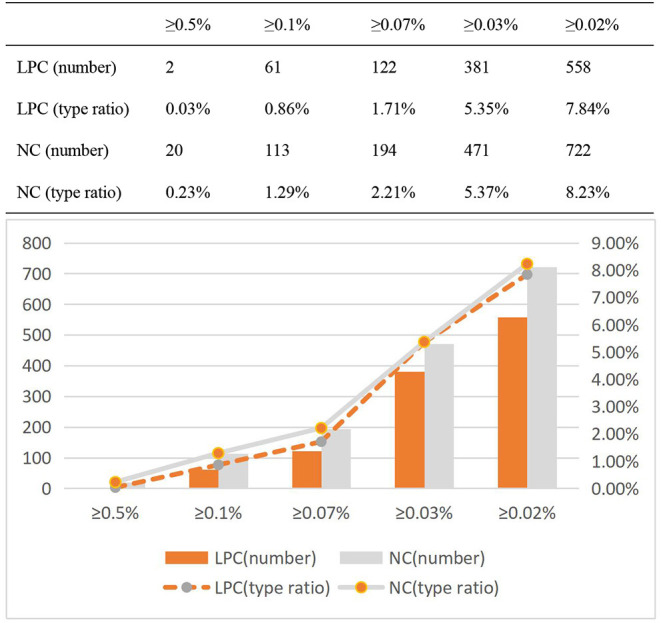
Type ratio distribution of high frequency words of LPC and NC.

Compared with ultra high frequency words and high frequency words, NC's secondary high frequency words were significantly higher than LPC's. Therefore, the token ratio of ultra high frequency words, high frequency words and secondary high frequency words in the two corpora follows a certain rule, but there are also some differences. Now, the distribution information of type ratio of high frequency words of LPC and NC is analyzed, as shown in Figure 6.

As shown in Figure 6, the type ratio of high frequency words of English NC is higher than that in Chinese LPC in all categories. But unlike the token ratio, the type ratio curves of the two are basically similar, and even almost identical in secondary high frequency words (≥0.03% of total corpus). There are only slight differences between them in ultra high frequency words and high frequency words, but no significant differences in the whole. The difference in the number of secondary high frequency words (≥0.02% of total corpus) between them is the largest.

The research shows that compared with Chinese LPC, English NC uses more secondary high frequency words with more types, while the Chinese LPC tends to use fewer high frequency words, which further illustrates the influence of different texts on the use of language vocabulary. This study discusses the distribution of high frequency words, and adopts the method of observing the number of high frequency words, token-type ratio and the change of the ratio, which is helpful to understand the influence of the quantitative dimension of language index on the description of different types of text. But, in the legal text and news text, it is the content words that play the main semantic role. The study of the token-type distribution of high frequency words alone is not enough to explore whether the focus and concern of the two corpora are consistent. Therefore, the next section will compare the high frequency content words and their collocations of the two corpora in order to discuss whether the focus and value of the two corpora are consistent.

### High Frequency Content Words and Their Collocations

According to AntConc 3.4.4w, the content words ranked top eleven in frequency in the two corpora are shown in Table 9.

**Table 9:** Content words ranked top eleven in frequency in the two corpora.

**LPC**		**NC**	
**Word**	**Frequency**	**Word**	**Frequency**
发展 (development)	801	China	1,614
企业 (enterprise)	756	Said	1,117
服务 (service)	538	Development	501
疫情 (COVID-19)	517	Global	466
建设 (construction)	507	World	444
工作 (job)	487	Economic	440
经济 (economy)	486	Market	420
推进 (improvement)	463	Growth	355
改革 (reform)	414	International	350
政策 (policy)	387	Cooperation	284
管理 (management)	341	Recovery	263

Table 9 shows that “development” is the most high frequency word in the two corpus (excluding proper nouns and verbs that have no specific meaning). It can be seen that “development” is the focus of economic laws, policies and economic news in China during the COVID-19 pandemic. Due to the COVID-19 pandemic, the world economy has been affected, and economic development has naturally become the focus of the economic field, which is clearly reflected in China's economic laws, policies and news during this period. “Development” is a relatively abstract concept, as a symbol, it has the feature of temporality and spatiality, that is, it has different forms of expression and meaning in different fields and periods. In addition, it can also be found that the word “said” has a high frequency in the news text. In this study, the concordance function of AncConc 3.4.4w was used to investigate the collocations of the word “development” in the two corpora, so as to more comprehensively understand its expression meaning, as shown in Table 10.

**Table 10:** Collocations of “development” in the two corpus.

**LPC**	**NC**
经济社会发展 (economic and social development)	Stage of development
规划发展 (planning and development)	Development stage
发展战略 (development strategy)	Development area
改革发展 (reform and development)	Investment and development
发展要求 (development requirement)	Global development
企业发展质量 (enterprise development quality)	New technology development
发展定位 (development orientation)	Boosting regional development
发展目标 (goal of development)	Development for recovery
发展的原则 (principles of development)	Development of high-tech
发展理念 (concepts for development)	Development in Shanxi
协调发展 (coordinated development)	Development in China
发展计划 (development planning)	Development and cooperation
高质量发展政策 (high-quality development policies)	Development of foreign trade
发展规范 (development norm)	Sustainable development
发展规划纲要 (outline of development plan)	Development and prosperity
加快发展 (speed up development)	Development of China's film
发展氛围 (atmosphere of development)	Achieving development

Table 10 shows that there are differences in development perspectives between the Legal Policy Corpus and the News Corpus. For example, LPC pays more attention to the development at the macro level, locating and naturalizing the whole development from the direction, plan and other aspects; NC, on the other hand, pays more attention to the development at the micro level, showing the concrete achievements and practices of development from the practical perspective. Even though “development” is mentioned by both, the legal policy plan for development, while the news focuses on the results and effects of development.

## Discussion

### Word Length Features of LPC and NC

Word length is one of the effective standards to measure the difficulty of text and the complexity of language units. Zipf [(32), p. 38] discovered the relationship between the occurrence frequency of a word in a text and its frequency rank (ordinal number), and proposed Zipf's law. Wang (44), Deng & Feng (33), Chen (45) conducted correlation statistics on word length and word frequency, which contributed evidence to relevant theories. In this study, word length is also used as the research index of lexical text characteristics. The results show that word length and frequency of the two corpora are influenced by language and style to a certain extent, and the relationship between them accords with the economic principle of language on the whole. The more letters in a word, the fewer words there are.

Kendall's tau-b grade correlation coefficient test can also prove that LPC is significantly correlated with NC word length distribution (*p* < 0.001) (Table 4). (46) believed that disyllabic words are the most common verbs in modern Chinese, while monosyllabic words are mostly action verbs. There are more 2-letter words in Chinese, followed by 1-letter words, while there are more multi-syllable words in English, which accords with the main characteristics of the distribution of disyllable word length in Chinese and English. As can be seen from Figure 1, with the increase of word length, the number of words in the two corpora decreases as a whole, which can reflect that the word length distribution of the two corpora basically conforms to the Principle of Least Effort.

But different data are different in function distribution and matching degree, curve fitting is one of the basic methods in experimental data processing. In this study, curve fitting is used to discuss the distribution of word length and frequency of the two corpora. Gong [(47), p. 73] conducted a functional curve analysis of the distribution of word length and frequency in the study of legal translation visibility, and believed that Chinese texts were more in line with the power function distribution, while English texts were more in line with the logarithmic and exponential function distribution. However, this study shows that English texts do not conform to the logarithmic function distribution, but only to the exponential function distribution, which also reflects that different genres (law and news) have certain influence on the distribution characteristics of word length and frequency.

### Word Cluster Characteristics of LPC and NC

Word cluster has both lexical and grammatical features and can reflect the characteristics of language repetition. It can be stored and used as a whole (48), thus virtually reducing the burden of language processing and output and making language communication more efficient, fluent and effective (49). Biber (50), Cortes (51), and other scholars analyzed the high frequency word clusters in discourse. Now, the study of word clusters is one of the main fields of corpus linguistics, and they regard word clusters as the meaning units in language (52, 53).

In this study, word cluster is used as the index of lexical text repetition rate. The results show that the frequency trend of word clusters in the two corpora is basically the same, the more words in the word cluster, the less of the word cluster.

Compared with English news texts, Chinese legal texts tend to use longer word clusters, and repetitive language fragments are more likely to occur. To a certain extent, legal policies are more standardized and rigorous than news, requiring the approval of state organs at all levels and certain normative requirements for the overall macro situation. Therefore, specific words or phrases, such as specific institutions, policies and regulations, are more often used. News language is relatively free, and the reader is people at all levels. Therefore, the language is more lively and more simple, and short words are used to make it easier for readers to accept it quickly.

### Distribution Characteristics of High Frequency Words of LPC and NC

Word frequency refers to the number of occurrences of specific words in a text, which to some extent reflects the stylistic characteristics of a text and is an important reference index for discovering stylistic or stylistic characteristics (54). High frequency words are often used in corpus research, which can reflect the integration of text to information resources and text focus. Johansson (55) compared and analyzed the 50 words with the highest word frequency in the corpus of academic text and fiction text, and found that some words belong to the corpus of academic text, while some specific words belong to the corpus of fiction text, which indicates that the high frequency words are different with different linguistic contexts.

Gong [(47), p. 89] combined with previous studies, discussed the distribution of high frequency words and rare words, adopted the method of mutual evidence of the difference between the proportion changes of token ratio and type ratio, and believed that high frequency words are related to the translation. This paper also discusses the distribution of high frequency words in the two corpora by observing high frequency word type ratio and token ratio, and finds that type ratio and token ratio of high frequency words in English NC are significantly higher than that in Chinese LPC.

In the aspects of ultra high frequency word (≥0.5% of total corpus), high frequency word (≥0.07% of total corpus), high frequency word (≥0.05% of total corpus), secondary high frequency word (≥0.03% of total corpus) and secondary high frequency word (≥0.02% of total corpus), Chinese LPC is lower than English NC. Based on the statistics of the distribution information of token ratio and type ratio of high frequency words of the two corpora, this study finds that, compared with Chinese LPC, English NC tends to use secondary high frequency words with rich types. The legal policy corpus use fewer high frequency words, which further demonstrates that high frequency words are also associated with different text categories or genres.

“From the distribution features of semantic categorization, verbs related to psychology, life activities and social activities are used more frequently” (46). There are few changes of morphology and vocabulary in Chinese, and the language of legal policy is less flexible than that of news. The news language covers a wide range, and there are many changes in English tenses and forms, which have a certain influence on the distribution of high frequency words. “Analysis of word frequency distribution shows that functional words in news headlines are often omitted. No frequent use of first and second person pronouns, the word ‘say' is frequently used and the words of news have the characteristics of the times (54). Therefore, high frequency words are not only influenced by translation, but also by different languages, styles or genres.

### Interpretation of High Frequency Content Words and Their Collocation in LPC and NC

Since, Firth [(56), p. 12] first put forward the concept of lexical collocation, Halliday, Sinclair and other scholars have carried out a series of pioneering researches on the definition of collocation and related terms: [(57), p. 284–87] put forward the concept of collocation and cohesion in a text, believing that the collocation of words is the function of cohesion in a text. Leech [(58), p. 17] put forward the Collocation Semantic Theory and defined the collocation semantic meaning of words as the relevant meaning obtained from the meaning of context words. Li (59) discussed the collocation characteristics of grammatical words and content words in the report and the address, and found that the two collocation frames are quite different, and the collocation frames with the same form have different semantic trends and pragmatic functions.

The study of high frequency words and their collocation features is also reflected in the field of legal language, Cheng and Pei (60) makes a semiotic explanation of the high frequency words and their collocation in network security law, and believes that legal terms are a kind of symbol with the characteristics of temporality and spatiality. Hu and Qian (61) compared and analyzed the interaction and mutual influence between language and politics in the “Work report of the Chinese Government” and the “State of the Union Address of the United States” based on the collocation features of “develop.”

In this study, the high frequency content words in the two corpora are counted, and the data showed that the frequency of “development” was the highest in both corpora. During the COVID-19 period, the economy was affected by the COVID-19 pandemic. “During the Spring Festival alone in 2020, 78% of the catering enterprises lost 100% of their income, 80% of the wholesale and retail industry stagnated, and the entertainment and tourism industry declined by about 85% compared with the same period last year” (62). The outbreak of large-scale pandemic not only seriously threatened public health security, but also had a significant impact on the stable development of the economy. The outbreak has led to a halt in economic activities, leading to economic recession, which can only be alleviated by the resumption of economic activities (i.e., economic development) after the outbreak is under control. Therefore, “the policy of pandemic prevention and control is also the policy of economic recession mitigation” (63). Therefore, economic development is the focus of economic laws, policies and news during the pandemic.

According to the statistics of high frequency content words, the word “said” appears frequently in news corpus, which is in pursuit of timeliness and authenticity. “Said” can directly reflect the interviewees' original words and attitudes, increase the authenticity and credibility of news, and at the same time give news text the characteristics of simplicity and directness of vocabulary. On the contrary, legal policies need to be checked by governments at all levels or relevant personnel, and the language needs to be highly normative. Personal subjective words are strictly prohibited, so related words such as “said” which are optional will not be used.

In terms of the collocation of high frequency word “development,” the two have different standpoint, which reflects their different perspectives of development. The fuzziness, accuracy and normality of legal discourse are its three main characteristics. In legal policies, there are often appears the word “idea,” “required” and others which can fully guarantee legal language extension of concept and expand to the scope of the law requires it, and “improve the flexibility and applicability of the law, make up of a series of problems brought by the legal lag, provided judges greater discretion, so as to achieve judicial justice as far as possible” (64). This is also reflected in the results of this study. LPC pays more attention to the development at the macro level, and the words that collocated with “development” are mostly “plan,” “positioning,” “goal,” “requirement,” “strategy” and other words for the overall situation. The aim is to provide a framework for economic development that can be referenced and standardized in countries affected by the pandemic, to provide a development framework for relevant economies (such as enterprises and factories), and to make plans for national economic recovery.

Compared with the tedious and formal written language of legal policy, “colloquialism is more and more easy to understand and accept in news” (65). Nowadays, “news reports play an important role of spiritual guidance and cultural guidance, which requires news language to be rational and consistent with national policies, considering the needs of the public, and expressing in the way of public thinking” (66). The results of this study also reflect this. NC pays more attention to the development at the practical level, and starts with the policy implementation effect and the real-time overview of economic recovery to display the domestic economic development. In terms of collocation words, NC uses more vivid words such as “stage,” “area,” “recovery” and “high-tech,” or analogies or extensions, to report the actual economic development results.

Although they have different perspectives, they share a positive attitude toward economic development. In different ways, the two show the importance of national economic recovery and hold a positive attitude toward it. National economic policies have actively set the development direction for the economies of the affected countries during the pandemic period, targeted the development of economies at all levels, formulated development goals and plans, and actively promoted national economic recovery. Economic news reports are objective and fair, keeping pace with national policies and truly showing the domestic economic development under national policies. At the same time, the news also gives full play to its advantages of international communication, actively responds to the requirements of national development strategy and enterprise development positioning, makes use of its own platform, actively appeals for international cooperation and investment, and objectively presents the real economic situation of China on the international level.

## Conclusion and Implication

By means of corpus analysis, this paper finds that English economic news texts and Chinese economic legal policy texts share certain commonalities and also have some differences in the distribution of word length, word frequency, word cluster and the collocation of high frequency words, which reflects certain synchronicity between legal policy and news.

First of all, the distribution of word length, word frequency, word clusters and high frequency words are influenced by language and style to a certain extent. The word length and frequency of Chinese words is more in line with the power function distribution, while the word length and frequency of English words is more in line with the exponential function distribution. Due to the difference in genre characteristics between news and law, compared with news texts, legal texts tend to use longer word clusters, and the frequency trend of word clusters is basically the same between them. The feature of word length, word frequency and word cluster of legal policy texts and English texts accord with the economic principle and Principle of Least Effort of language as a whole. The distribution characteristics of high frequency words are also different depending on the genre and language. The type ratio and token ratio of high frequency words in English news texts are significantly higher than those in Chinese legal policy texts, and English news tends to use secondary frequency words with rich types and strong activity.

Secondly, through the analysis of the high frequency content words and their collocations, it can also be found that during the pandemic period, “development” is the focus of China's economic legal policies and economic English news. During the pandemic, the economy was affected by the COVID-19 pandemic. To mitigate the economic recession are an important issue of concern for economic legal policies, and the news provides live coverage of the benefits and results of economic policies. In different ways, the two show the importance of national economic recovery and hold a positive attitude toward it. The state has formulated reasonable policies and economic laws for the recovery of affected economies and actively promoted their economic recovery. The news media platform plays its role of international publicity through its advantages of wide dissemination and reader, and actively appeals for international cooperation and investment to show the reality of China's economy. This fully reflects the synchronization of China's economic news and economic legal policies in the field of concern, and maintains a positive and objective attitude toward the economic development during the COVID-19 pandemic.

This study combines the study of lexical features with the study of high frequency word collocation to conduct a macroscopic investigation of internal structure and external environment, thus enriching the study of linguistic lexical features and linguistic sociocultural features, and provide some references for relevant research. Although some differences and commonalities between Chinese legal texts and English news texts can be understood through the comparison of lexical features and collocations, more problems of linguistic complexity and relevance between them need to be further explored.

## Data Availability Statement

The original contributions presented in the study are included in the article/supplementary material, further inquiries can be directed to the corresponding author/s.

## Author Contributions

The author confirms being the sole contributor of this work and has approved it for publication.

## Conflict of Interest

The author declares that the research was conducted in the absence of any commercial or financial relationships that could be construed as a potential conflict of interest.

## Publisher's Note

All claims expressed in this article are solely those of the authors and do not necessarily represent those of their affiliated organizations, or those of the publisher, the editors and the reviewers. Any product that may be evaluated in this article, or claim that may be made by its manufacturer, is not guaranteed or endorsed by the publisher.
